# Supplementary figures and images for: Copper Is Accumulated as Copper Sulfide Particles, and Not Bound to Glutathione, Phytochelatins or Metallothioneins, in the Marine Alga Ulva compressa (Chlorophyta)
Source: Int J Mol Sci. 2024 Jul 11;25(14):7632. doi: 10.3390/ijms25147632 (PMC11277147; doi:10.3390/ijms25147632)

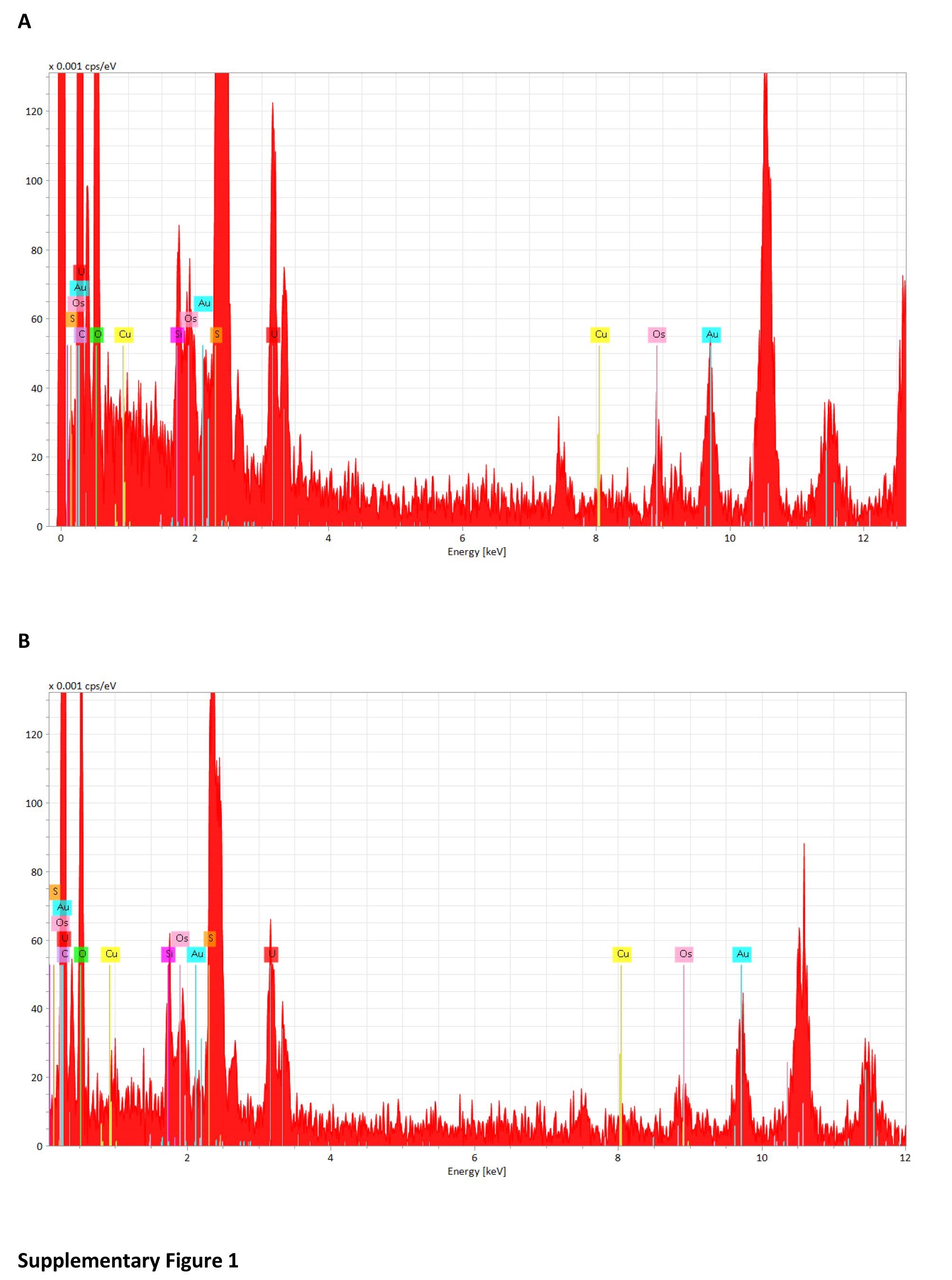

Supplement: Supplementary file 1 [file ijms-25-07632-s001.zip › ijms-3090776-supplementary.tif]
